# Regulatory role of microRNA in mesenteric lymph nodes after *Salmonella* Typhimurium infection

**DOI:** 10.1186/s13567-018-0506-1

**Published:** 2018-02-01

**Authors:** Juber Herrera-Uribe, Sara Zaldívar-López, Carmen Aguilar, Cristina Luque, Rocío Bautista, Ana Carvajal, M. Gonzalo Claros, Juan J. Garrido

**Affiliations:** 1 Grupo de Genómica y Mejora Animal, Departamento de Genética, Facultad de Veterinaria, Universidad de Córdoba, 14047 Córdoba, Spain; 2 Plataforma Andaluza de Bioinformática, Universidad de Málaga, 29590 Málaga, Spain; 3 Departamento de Sanidad Animal, Facultad de Veterinaria, Universidad de León, 24071 León, Spain; 4 Departamento de Biología Molecular y Bioquímica, Universidad de Málaga, 29071 Málaga, Spain; 5 Present Address: Institute for Molecular Infection Biology, University of Würzburg, Würzburg, Germany

## Abstract

Salmonellosis is a gastrointestinal disease caused by non-typhoidal *Salmonella* serovars such as *Salmonella* Typhimurium. This pathology is a zoonosis, and food animals with subclinical infection constitute a vast reservoir for disease. After intestinal colonization, *Salmonella* Typhimurium reaches mesenteric lymph nodes (MLN), where infection is controlled avoiding systemic spread. Although the molecular basis of this infection has been extensively studied, little is known about how microRNA (miRNA) regulate the expression of proteins involved in the *Salmonella*-host interaction. Using small RNA-seq, we examined expression profiles of MLN 2 days after infection with *Salmonella* Typhimurium, and we found 110 dysregulated miRNA. Among them, we found upregulated miR-21, miR-155, miR-150, and miR-221, as well as downregulated miR-143 and miR-125, all of them previously linked to other bacterial infections. Integration with proteomic data revealed 30 miRNA potentially regulating the expression of 15 proteins involved in biological functions such as cell death and survival, inflammatory response and antigenic presentation. The inflammatory response was found increased via upregulation of miRNA such as miR-21 and miR-155. Downregulation of miR-125a/b, miR-148 and miR-1 were identified as potential regulators of MHC-class I components PSMB8, HSP90B1 and PDIA3, respectively. Furthermore, we confirmed that miR-125a is a direct target of immunoproteasome component PSMB8. Since we also found miR-130 downregulation, which is associated with upregulation of HSPA8, we suggest induction of both MHC-I and MHC-II antigen presentation pathways. In conclusion, our study identifies miRNA that could regulate critical networks for antigenic presentation, inflammatory response and cytoskeletal rearrangements.

**Electronic supplementary material:**

The online version of this article (10.1186/s13567-018-0506-1) contains supplementary material, which is available to authorized users.

## Introduction

Salmonellosis is a major public health concern caused by Gram negative bacteria belonging to the family *Enterobacteriaceae* [1]. Non-typhoidal *Salmonella* serovars such as Typhimurium are the causative agents of food poisonings world-wide. They are usually transmitted to humans by eating contaminated animal-derived food products, mainly poultry, eggs, and pig products [2]. *Salmonella* Typhimurium is among the most commonly isolated food borne pathogens associated with pig and pork meat [3]. Although pigs may carry the bacterium without exhibiting clinical signs, *Salmonella* Typhimurium is the cause of significant animal suffering and important economic losses to the swine industry [3]. In addition, asymptomatic carrier animals spread the pathogen within herds, representing a point of entry into the human food chain. Thus, salmonellosis control in swine production is necessary for both public and animal health.

After *Salmonella* invades intestinal tissues, it reaches the mesenteric lymph nodes (MLN), which play an important role in immune defense against intestinal bacterial pathogens [4, 5]. To prevent systemic infection, MLN form a life-saving firewall that protects the host from rapid pathogen dissemination beyond the intestine. *Salmonella* Typhimurium remains for a long time in mouse MLN, establishing a persistent infection in the host [6]. In pigs, persistence of *Salmonella* Typhimurium in MLN has been reported up to 6 weeks after oral inoculation, sustaining these organs as major inductive sites for immune responses during porcine salmonellosis [7]. It has been shown that *Salmonella* Typhimurium expresses some of its major virulence effectors in porcine MLN [8]. In spite of that, a combination of early innate and adaptive immunity mechanisms overcome virulence strategies used by the pathogen, enabling the host to protect itself against bacterial spread beyond gut-associated lymph nodes [8, 9].

miRNA are small, non-coding RNA that regulate gene expression post-transcriptionally through complementary binding to sequences in the 3′untranslated region of target mRNA, resulting in translational inhibition [10]. After bacterial infection, miRNA regulate host responses by modulating the expression of genes involved in a variety of cellular processes such as proliferation, cell growth, cell death, inflammation and development [11, 12]. Some in vitro and in vivo studies have reported altered miRNA expression following *Salmonella* infection. Thus, in macrophages and epithelial cells, *Salmonella* Typhimurium affects expression of miR-21, miR-146a, miR-155, let-7 and miR-15, which regulate genes involved in inflammatory response, T and B cell proliferation and cell cycle [13]. Furthermore, miRNA can modulate the response to *Salmonella* infection in pigs. For instance, miR-29a and miR-128 upregulation during infection is related to the control of the intestinal epithelial cell proliferation and a decrease in the recruitment of macrophages in the ileal mucosa, respectively [14, 15]. Another study in porcine whole blood showed changes in the expression of miR-124 and miR-331-3p, whose target genes encode for proteins associated with the regulation of the immune response [16]. There are very few studies about the function of miRNA in innate and adaptive immunity of porcine lymph nodes, most of them related to viral infections [17, 18]. Given the pivotal role of the MLN in the control of *Salmonella* Typhimurium infection in pigs [8], the aim of the present study was to characterize miRNA expression differences in porcine ileocecal MLN upon *Salmonella* challenge. In addition, to obtain a more accurate prediction of miRNA targets than those provided by bioinformatic tools, miRNA expression data were integrated with our previous large-scale protein expression data to identify immune response-related miRNA. To our knowledge, this is the first time that the regulatory role of miRNA in gene expression is studied by integrating protein and miRNA expression data in order to provide a more comprehensive view of the immune response to *Salmonella* Typhimurium infection in pigs.

## Materials and methods

### Experimental infection and sample processing

The experimental infection design was described elsewhere [19]. Briefly, eight male and female crossbred weaned piglets, approximately 4 weeks of age, were used in this study. All piglets were derived from a *Salmonella*-negative herd and were serologically negative before the experiment. Four piglets were necropsied 2 h prior to experimental infection [control group, 0 days post-infection (dpi)]. The 4 remaining piglets were challenged orally with 10^8^ colony forming units (cfu) of a *Salmonella* Typhimurium phagetype DT104 strain isolated from a carrier pig [20] and necropsied at 2 days post infection (infected group, 2 dpi). Ileocecal MLN samples were collected from all animals immediately after euthanasia and frozen in liquid nitrogen for further RNA isolation. For bacteriological culture, fecal samples were collected prior to challenge and immediately before animals were sacrificed. Fecal sample processing and bacteriological analysis were performed following the current EN-ISO standard methodology 6579:2002/Amd 1:2007. All infected animals were positive for *Salmonella* Typhimurium in feces prior to their necropsy, whereas control animals were negative. All procedures involving animals were previously approved by the institutional bioethical committee, and performed according to European regulations regarding animal welfare and protection of animals used for experimental and other scientific purposes.

### RNA isolation

Total RNA from MLN samples were isolated using mirVana miRNA isolation kit (Ambion Inc, Austin, TX, USA). Eluted RNA was treated with DNase using TURBO DNA-free™ Kit (Ambion Inc) to eliminate traces of DNA. RNA integrity was assessed in the Agilent 2100 Bioanalyzer (Agilent Technologies, Palo Alto, CA, USA). Only samples with RNA integrity numbers (RIN) ≥ 7 were used for further analysis.

### Small RNA library preparation and next generation sequencing

Pooled RNA samples containing two individuals were randomly composited by blending equal concentrations of each RNA. Then, four pooled RNA samples (2 controls and 2 from infected animals) were used for small RNA sequencing (small RNA-seq). Library preparation and sequencing were performed at the Functional Genomics Core of the Institute for Research in Biomedicine (IRB Barcelona). Five hundred nanograms of total RNA per sample were used for library preparation using the NEBNext^®^ Multiplex Small RNA Library Prep Set for Illumina (New England Biolabs Inc, Ipswich, MA, USA). Libraries were quantified with Qubit dsDNA HS assay (Thermo Fisher Scientific Inc, Waltham, MA, USA) and quality was assessed using an Agilent 2100 Bioanalyzer (Agilent Technologies). Each library was sequenced on a HiSeq2000 (Illumina Inc., San Diego, CA, USA) using a 50 bp single-end reads’ sequencing strategy. All sequences were deposited at NCBI Sequence Read Archive (SRA) with accession number SRP110581.

### Sequencing data analysis

Bioinformatic analysis was performed at the Andalusian Platform of Bioinformatics of the University of Malaga. Raw reads were pre-processed using the in-house developed customizable pre-processing pipeline SeqTrimNext [21]. Contaminants, sequencing adapters, short (< 17 nucleotide) and bad quality reads (Phred score < 20) were removed, to ensure that only high quality sequences were used for further analyses. Reads were mapped against the reference databases Rfam (version 11.0), miRBase (v20), Human Genome Assembly (GRCh37) and Swine Genome Assembly (susScr3). The CAP-miRSeq pipeline was applied to identify porcine miRNA and calculate their expression [22]. Only differentially expressed (DE) miRNA with a corrected *P* value < 0.05 were considered for further investigations.

### Quantitative real-time PCR (qPCR)

All expression analyses were performed following the “Minimum Information for Publication of Quantitative Real-Time PCR Experiments (MIQE)” guidelines [23]. For quantification of gene expression, 1 μg of total RNA was reverse-transcribed using qScript™ cDNA synthesis kit (Quanta Biosciences Inc., Beverly, MA, USA), according to the manufacturer’s instructions. Real-time quantitative analysis was performed using a QuantStudio 12 K system (Applied Biosystems Inc., Foster City, CA, USA). The 10 μL PCR reaction included 1 μL of 1:5 diluted cDNA as template, 2 μL of 5× PyroTaq EvaGreen qPCR Mix Plus with ROX (Cultek Molecular Bioline, Madrid, Spain), and 20 μM of transcript-specific forward and reverse primers (Additional file 1). Thermal cycling conditions included an initial denaturation at 95 °C for 5 min, followed by 35 cycles of 30 s at 94 °C, 30 s at 57 °C and 45 s at 72 °C. For miRNA quantification, 100 ng of total RNA were reverse transcribed to cDNA as previously reported [24] and diluted eightfold. miRNA-specific primers were designed according to guidelines set by Balcells et al. [24] using publicly available software miRprimer [25] (Additional file 1). A 10 µL PCR reaction was used containing 1 µL of cDNA, 2 µL of 5× PyroTaq EvaGreen qPCR Mix Plus with ROX (Cultek Molecular Bioline, Madrid, Spain), and 10 µM of each primer. Cycling conditions were 10 min at 95 °C followed by 40 cycles of 5 s at 95 °C, and 60 s at 60 °C. Melt-curve analyses were performed immediately after the amplification protocols to ensure single size amplicon production. Relative gene and miRNA expression were calculated using the 2^−ΔΔCt^ quantitation method (GenEx software, MultiD Analyses AB, Göteborg, Sweden) [26]. Expression stability of the candidate reference genes and miRNA was calculated by the NormFinder and geNorm programs [27, 28]. Gene expression data were normalized to β-2 microglobulin (*B2M*) and cyclophilin A genes (*PPIA*), whereas let-7a, miR-26a, miR-16-5p, miR-103 and miR-17-5p were used to normalize the miRNA expression data. Statistical differences in expression values among groups were assessed using the Student’s *t* test. Additionally, a Pearson correlation analysis was performed to validate small RNA-Seq data by qPCR (Graphpad Prism 6, Graphpad Software Inc, La Jolla, CA, USA). The significance level was set at *P* < 0.05.

### Target gene and integrative analysis

Previous proteomic expression data [9] were integrated with the DE miRNA to scan for potential target genes. TargetScan (release 7.0) [29] and miRTarbase (release 6.0) [30] were used to detect predicted miRNA target genes based on seed complementarity (on 3′-, 5′-UTR and coding sequences of the porcine mRNA) and reported experimental verification (i.e. reporter assay, western blot or qPCR). Only targets that were inversely expressed (miRNA upregulated, protein downregulated and vice versa) were selected for functional analysis.

### Systems biology analysis

Functional analysis of miRNA targets was carried out using Ingenuity Pathway Analysis (IPA, Ingenuity Systems^®^ Inc, Redwood City, CA, USA). Most significant biological functions (top 5, *P*-value ≤ 0.05) were selected to elucidate the role of miRNA in MLN from *Salmonella* Typhimurium infected animals.

### Identification of MRE in 3′UTR gene region

miRNA recognition elements (MRE) were predicted with TargetScan and RNAhybrid programs [29, 31]. TargetScan prediction criteria is based on strong pairing in the seed region, thermodynamic stability, number of target sites on the 3′UTR of a given mRNA, sequence context (location of MRE with respect to stop codon and to the poly A tail or presence of AU-rich clusters) and accessibility of the target site to the RISC complex [32]. The hybridization energy required for the formation of miRNA-MRE duplex was calculated by uploading to RNAhybrid the sequence of 3′UTR segments containing the MRE and their respective miRNA. Only the duplexes with favorable hybridization energy of ≥ −15 kcal/mol were chosen as potential MRE.

### miRNA target validation

The 3′UTR of *PSMB8* and *PSMB9* containing the miR-125b-5p binding site were amplified by PCR using the following primers: GGTCTCGAGCCACTGGTAGCGGGAACACC (forward) and GGTCTCGAGCAACCCCAACCTTCTGGGCAT (reverse) for *PSMB8*, and GGTCTCGAGGGTTATGTGGACGCGGCATA (forward) and GGTCTCGAGAGGCCATCATGTTTTGAGTGATTT (reverse) for *PSMB9*. Each PCR product was cloned separately into the psiCHECK™-2 vector (Promega Inc, Madison, WI, USA). The Chinese hamster ovary cell line (CHO) was cultured in RPMI (Biowest, Riverside, MO, USA) supplemented with 10% of heat inactivated fetal calf serum (Gibco, Carlsbad, CA, USA) and 2 mM l-glutamine (Biowest) at 37 °C and with 5% CO_2_. miRNA mimic miR-125b-5p and mimic negative control #1 (Dharmacon Inc., Lafayette, CO, USA) were separately reverse transfected into CHO cells at a final concentration of 75 nM, using Viromer Blue transfection reagent (Lipocalix Inc., Germany). miRNA and transfection reagent were prepared according to the manufacturers’ protocols, and 2 × 10^4^ cells per well were seeded on 96-well plates at 37 °C with 5% CO_2_. After 24 h, cells were transfected again with 250 ng of the psiCHECK™-2 vector containing target genes’ 3′UTR using Lipofectamine 3000 transfection kit (Thermo Fisher Scientific Inc.). The cell line was incubated for 48 h, and then the Dual-Glo luciferase assay system (Promega Inc, Madison, WI, USA) and Varioskan Lux microplate reader (Thermo Fisher Scientific Inc.) were used to measure the quantity of firefly and Renilla luciferase. The firefly luciferase quantity was first normalized to Renilla luciferase quantity, and those ratios were then normalized to empty vector controls. Statistical differences in expression values among groups were assessed using a Student’s *t* test (Graphpad Prism 6, Graphpad Software Inc, La Jolla, CA, USA). Statistical significance was set at *P* < 0.05.

## Results

### miRNA profile (miRNAome) in the porcine ileocecal MLN

To determine the miRNA expression pattern in response to *Salmonella* Typhimurium in pigs, the miRNA transcriptome was characterized by small RNA-seq using ileocecal MLN samples collected at 0 and 2 dpi. High-throughput sequencing produced a total of 30.7 million short reads, 14.5 million of which (47.5%) passed quality control thresholds and were mapped onto the ncRNA region from reference databases. As observed in Additional file 2, alignment of the reads against the human genome resulted more efficient than porcine mapping. BLAST search of the clean sequencing reads against the Rfam database revealed that most of the identified sequences were miRNA (34.1%), followed by other non-coding RNA types such as snoRNA, rRNA and tRNA, which accounted for 11.8, 4.8 and 3.3% of the non-coding reads, respectively.

Mapped miRNA were used to decode the miRNAome of MLN from infected and non-infected pigs (Additional file 3). Only miRNA with at least one aligned read in either library were considered. When mapped to the human database, an average of 492 and 503 miRNA were obtained in control and infected pigs, respectively (Additional file 3A). The numbers of identified miRNA decreased to 267 and 276, respectively, when sequence reads were mapped to *Sus scrofa* (Additional file 3B). Most of these miRNA were annotated in humans and pigs, even though 53 miRNA were annotated exclusively in humans (e.g. miR-200 family, miR-223, miR-93, miR-25 and miR-147) whereas only 13 were annotated exclusively in pigs (e.g. miR-7134, miR-2320, miR-4334 and miR-1839) (Additional file 3C). The human miRNA annotation was used for further analysis, based on the higher number of sequence reads mapped to the human database, the assumption that the majority of miRNA sequences are conserved among species [33] and the higher availability of target gene prediction data in humans. Table 1 shows the highly expressed miRNA in control and infected porcine MLN.

**Table 1:** **Most abundant miRNA present in mesenteric lymph nodes of control and** ***Salmonella*** **Typhimurium infected pigs**

Mature miRNA	Control	Infected
	%	%
miR-21-5p	17.1	24.5
miR-143-3p	11.4	7.8
miR-126-3p	8.3	5.7
miR-26a-5p	6.2	6.5
miR-148a-3p	6.1	2.9
let-7f-5p	4.1	4.2
let-7g-5p	3.7	5.3
let-7a-5p	2.8	2.6
let-7i-5p	2.8	4.2
miR-99a-5p	2.6	2.7
miR-10a-5p	2.1	1.1
miR-100-5p	2.1	1.3
miR-199a-3p	1.5	1.3
miR-146b-5p	1.3	1.3
miR-191-5p	1.2	1.4
miR-24-3p	1.1	1.1
miR-181a-5p	1	1.3
miR-27b-3p	1	1
miR-30d-5p	1	–
miR-142-5p	0.9	–
miR-150-5p	–	1.4
miR-92a-3p	–	1.1

Most abundant miRNA (top 20) present in mesenteric lymph nodes of control and infected pigs. Average percentage of reads of each miRNA (%) was calculated with respect to total number of reads obtained in each group.

### Differential expression of miRNA in porcine MLN after *Salmonella* infection

**Figure 1:**
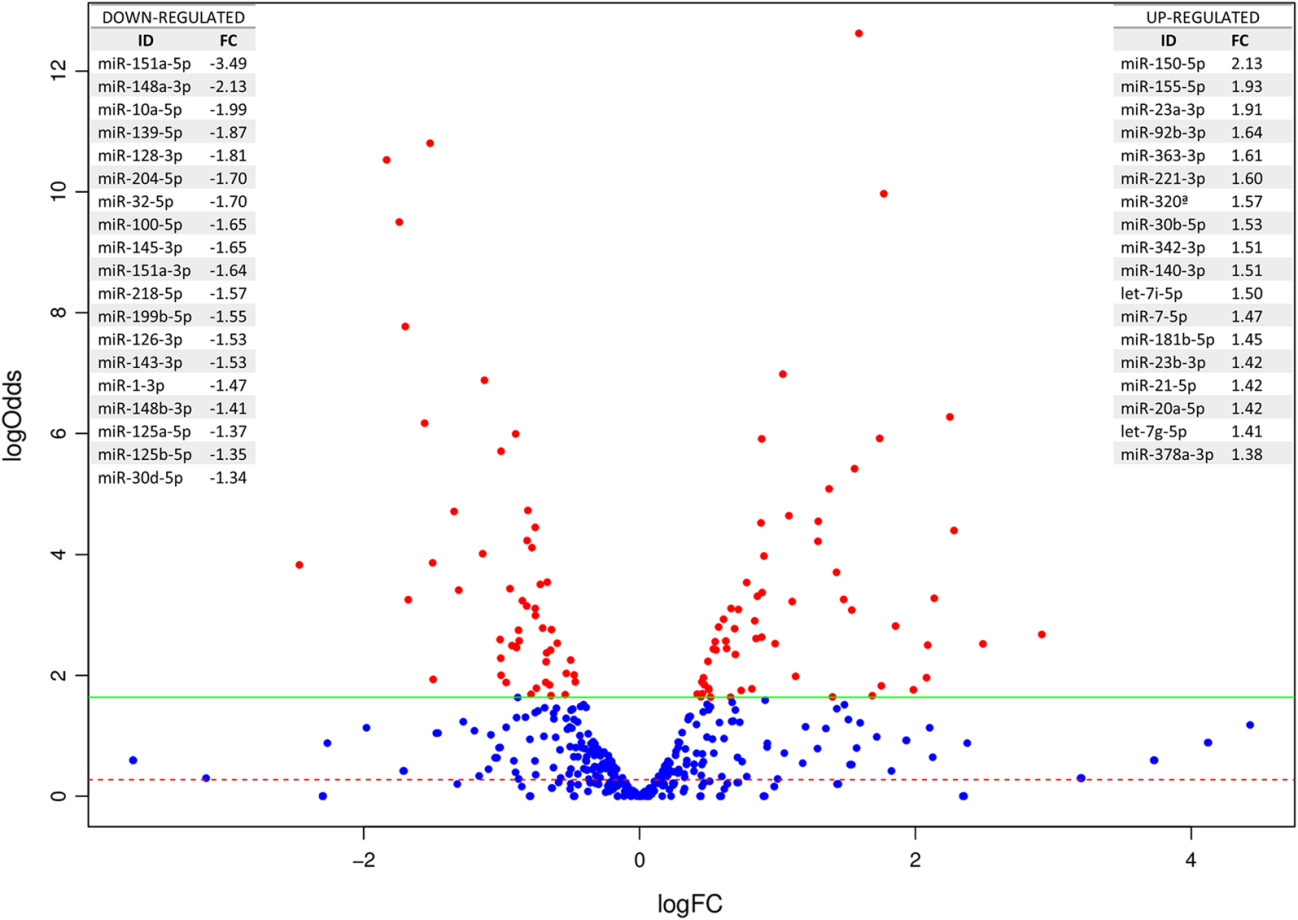
**Differentially expressed miRNA in infected MLN compared to non-infected controls.** Volcano plot showing differentially expressed miRNA in MLN compared to non-infected controls, highlighting in red those with a FDR-corrected *P*-value < 0.05. Left and right upper corner tables summarize most abundant (> 300 sequencing reads) down- and up-regulated miRNA, respectively.

**Figure 2:**
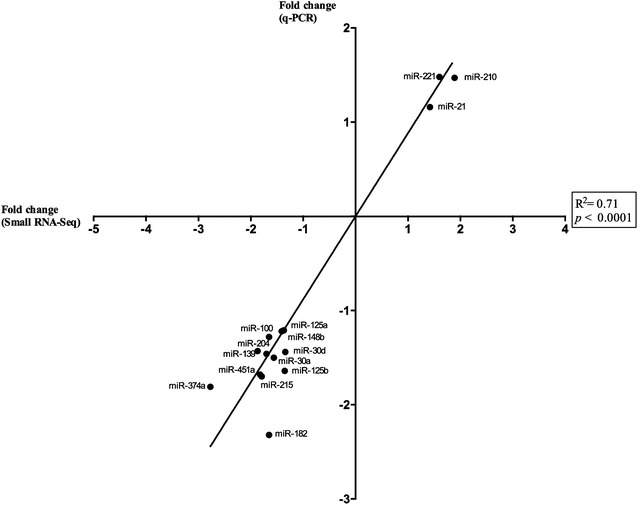
**Small RNA-seq result validation by correlation with qPCR.** Pearson correlation analysis between small RNA sequencing (small RNA-Seq) and quantitative real time PCR (qPCR) of 15 differentially expressed miRNA in mesenteric lymph nodes 2 days after infection with *Salmonella* Typhimurium.

Based on CAP-miRSeq analysis [22], 110 differentially expressed (DE) miRNA were identified in porcine MLN after *Salmonella* Typhimurium infection. Amongst those, 50 were downregulated (fold change—FC—ranging from −5.41 to −1.34) and 60 upregulated (FC ranging from 1.38 to 7.72) in the infected group compared to the control group (Figure 1, Additional file 4). Fifteen DE miRNA were selected for validation by qPCR based on the biological functions of their potential target genes. miRNA expression analyzed by qPCR was in good accordance with RNA-seq data (R^2^ = 0.71, *P* < 0.0001), confirming the reliability of the sequencing technique used in this work (Figure 2).

### Integrative analysis of miRNA and protein expression

**Figure 3:**
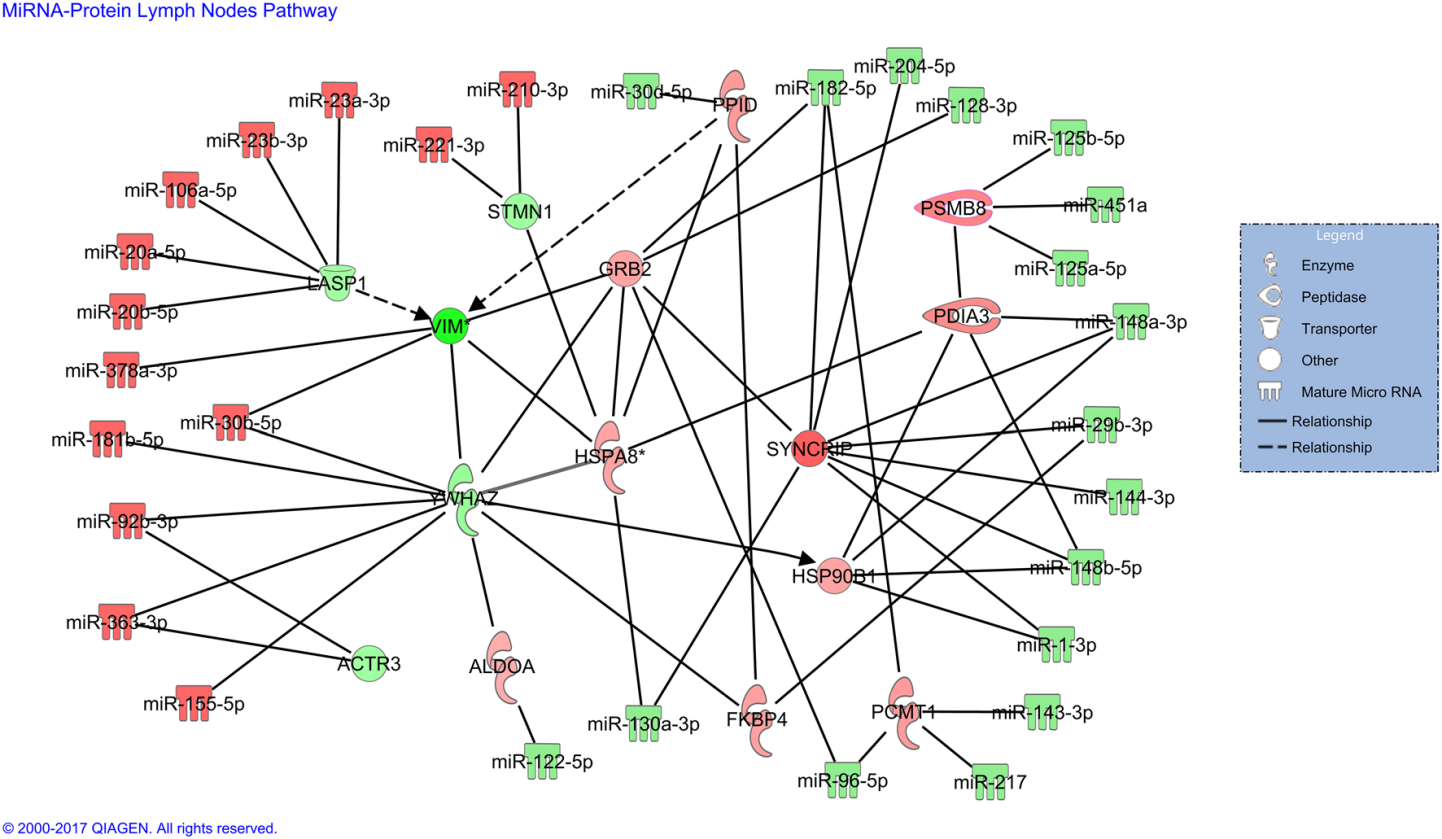
**miRNA-protein interaction network.** miRNA–protein interaction network resulting from the integration of small RNA-Seq and proteomic data of mesenteric lymph nodes at 2 dpi. Red: molecules over expressed; green: molecules down expressed.

TargetScan and miRTarbase softwares were used to predict target genes that could be regulated by the DE miRNA. In total, 39 366 miRNA–mRNA pairs were in silico predicted as functionally linked (Additional file 5). Then, integrative analysis was performed to evaluate associations between predicted target genes and the proteomic dataset previously identified by Martins et al. [9]. Only target genes encoding proteins shared with the historical dataset were included for further analysis (*n* = 98). Selected target proteins were matched and paired with DE miRNA in order to construct a functional miRNA–mRNA regulatory network. The miRNA–mRNA pairing strategy was based on the assumption that in most cases there is a negative correlation between miRNA and their target mRNA (miRNA upregulated/protein downregulated, and vice versa) [34]. miRNA–mRNA pairing resulted in 46 miRNA–protein interactions (Figure 3), suggesting that over-expression of 13 miRNA (miR-210-3p, miR-221-3p, miR-23a-3p, miR-23b-3p, miR-106a-5p, miR-20a-5p, miR-20b-5p, miR-378a-3p, miR-30b-5p, miR-181b-5p, miR-92b-3p, miR-363-3p, and miR-155-5p) could be regulating the down expression of 5 target proteins (STMN1, LASP1, VIM, YWHAZ and ACTR3). On the contrary, downregulation of 17 miRNA (miR-30d-5p, miR-182-5p, miR-204-5p, miR-128-3p, miR-125a-5p, miR-125b-5p, miR-451a, miR-148a-3p, miR-29b-3p, miR-144-3p, miR-148b-5p, miR-1-3p, miR-143-3p, miR-217, miR-96-5p, miR-130a-3p, and miR-122-5p) could be allowing the over-expression of 10 proteins (PPID, PSMB8, PDIA3, GRB2, HSPA8, SYNCRIP, HSP90B1, PCMT1, FKBP4, and ALDOA). To gain a deeper understanding of the functional alterations induced by DE miRNA, the constructed network was analyzed to determine the biological processes and molecular functions in which predicted target proteins were involved. We found that predicted target proteins were mainly associated with functions such as cell death and survival, cellular assembly and organization, inflammatory response and protein degradation (Table 2).

**Table 2:** **Biological functions affected by miRNA-regulated proteins in** ***Salmonella*** **Typhimurium infected mesenteric lymph nodes**

Biological functions: ordered by significance according to IPA results			
Category	−log (*P*-value)	# Molecules	Molecules
Cell death and survival	8.4	13	PPID, PDIA3, GRB2, PCMT1, YWHAZ, VIM, PSMB8, HSPA5, STMN1, HSPA8, HSP90B1, FKBP4, ALDOA
Cellular assembly and organization	5.7	12	PPID, HSPA8, STMN1, ACTR3, PDIA3, GRB2, YWHAZ, FKBP4, ALDOA, VIM, HSPA5, LASP1
Inflammatory response	5.5	10	PPID, HSPA8, HSP90B1, GRB2, PDIA3, FKBP4, ALDOA, VIM, PSMB8, HSPA5
Protein degradation	4.6	4	HSPA8, HSP90B1, PDIA3, HSPA5

Biological functions (*P* < 0.05) affected by differentially expressed proteins in mesenteric lymph node at 2 dpi potentially regulated by differentially expressed miRNA.

### Quantification of immune and cell death-related genes by qPCR

**Figure 4:**
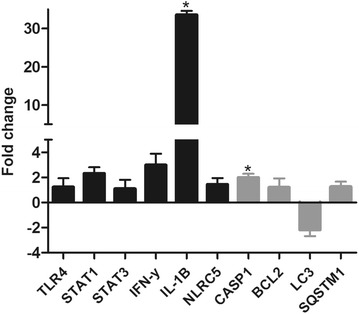
**Expression of genes involved in inflammatory response and cell death.** Expression of genes involved in inflammatory response (black) and cell death mechanisms (gray) in MLN samples at 2 dpi, compared to non-infected controls.

**Figure 5:**
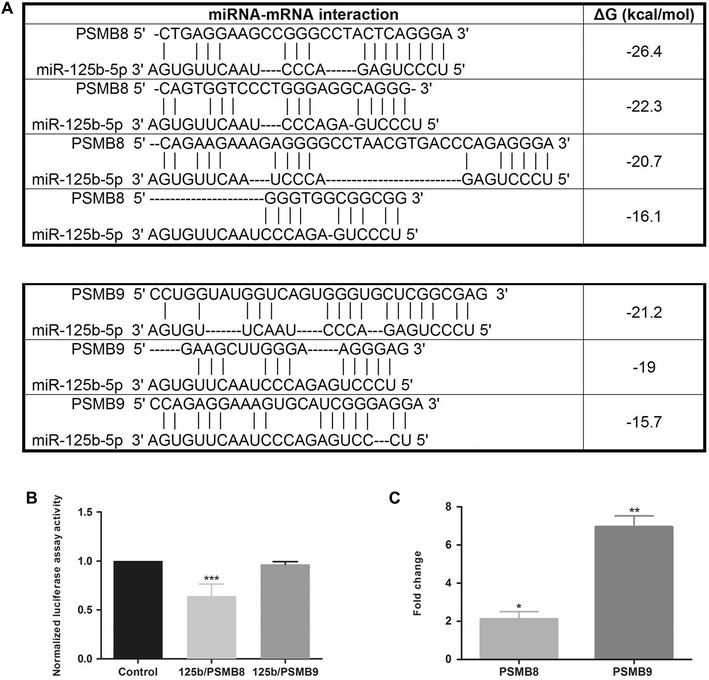
**Prediction analysis of miRNA-target interaction, results of luciferase assay, and gene expression of immunoproteasome components. A** Prediction of target sequence in miR-125b/PSMB8 and miR-125b/PSMB9 binding. The highest score obtained from RNAhybrid prediction (−15 kcal/mol, scoring criteria) was used for the design of the luciferase reporter assay. **B** Firefly luciferase activity was measured and normalized by the Renilla luciferase activity. **C** Gene expression of PSMB8 and PSMB9 in MLN samples at 2 dpi. Results are represented as mean ratio ± SEM from four independent transfection experiments. **P < 0.01 and *P < 0.05.

In order to confirm the alterations of these biological functions, gene expression profiling was performed for selected genes involved in inflammatory response (*TLR4*, *STAT1* and *STAT3*), antigenic presentation (*NLRC5*), cell death by pyroptosis (*IFN*-*γ*, *IL*-*1β* and *CASP1*), autophagy (*LC3* and *SQSTM1*) and apoptosis (*BCL2*). In general, we observed induction (over-expression) of all previously mentioned processes at 2 dpi, except for autophagy (Figure 4). Furthermore, we confirmed the over expression of *PSMB8* and *PSMB9* genes encoding two components of immunoproteasome (Figure 5).

### miRNA-target validation by luciferase reporter assay

As we demonstrated by small RNA-seq and qPCR, miR-125 was downregulated in porcine MLN after *Salmonella* Typhimurium infection. This miRNA is an important regulator of inflammatory response during *Salmonella* infection, given that miR-125 has been reported as target of TNF-α, and its downregulation is required for proper TNF-α production [35]. As we observed in the interaction network between DE miRNA and proteins, miR-125b-5p is predicted to interact with PSMB8 mRNA, which could indicate that this miRNA is a direct regulator of the PSMB8 protein expression. In addition, based on bioinformatic analysis, we predicted that miR-125b-5p interacts with other related target proteins such as PSMB9. Therefore, based on such integrative analysis and its biological implication during *Salmonella* Typhimurium infection (PSMB8 is involved in inflammatory response and cell death, Table 2), we designed a luciferase reporter assay to confirm whether PSMB8 and PSMB9 are direct targets of miR-125b. The miRNA-target interaction was tested by co-transfecting miR-125b-5p miRNA mimic with the vector construction containing the interacting 3′UTR of PSMB8 and PSMB9, separately, into CHO cells. Luciferase activity values obtained indicated a 31% downregulation of the luciferase activity when the miR-125b-5p mimic and PSMB8 were present (*P* < 0.01), but we did not find changes in luciferase activity values when the miR-125b-5p mimic and PSMB9 were present. Hence, these results confirm the direct interaction between miR-125b and the 3′UTR of the PSMB8 target gene, validating a previous bioinformatic prediction of the interaction between the sequences (Figure 5).

## Discussion

There is increasing evidence to suggest that miRNA are involved in the regulation of proteins involved in innate and adaptive immune pathways. However, despite numerous studies showing that miRNA are important in the host defense to infection, there is still little information regarding their role and mechanism of action. MLN play an essential role in protection against bacterial enteropathogens, notably acting as antigen sentinels of the gastrointestinal tract [36]. In pigs, MLN are key sites for antigen presentation in the adaptive immune response. However, in *Salmonella* infection, they also become niches of replication due to the ability of bacteria to survive in these organs [8]. Defense mechanisms in porcine MLN against *Salmonella* have been described by us previously. Here, we sought to determine the role of miRNA in regulating the pig response to *Salmonella* infection through the integration of miRNA and protein expression data and miRNA–RNA target prediction.

In our study, RNA-Seq was used to characterize the expression profile or miRNAome of porcine MLN after *Salmonella* Typhimurium infection. Previous studies have demonstrated that many miRNA are highly expressed in mammalian tissues and thus are thought to play important roles in biological functions [37, 38]. In this work, a total of 492 and 503 miRNA were identified in control and infected pigs, respectively. Some of the expressed miRNA such as miR-21 and miR-143 showed the highest level of expression in both experimental conditions, therefore suggesting that these miRNA are abundantly expressed in porcine MLN. miR-21 was found upregulated in infected pigs, which is consistent with increased abundance of miR-21 in inflammatory diseases [39], and suggests that this miRNA could play a significant role in the inflammatory response to *Salmonella*. In addition to miR-21, previous studies have revealed the roles of other DE miRNA such as miR-143, miR-155, miR-150, miR-221 and miR-125a/b in pathogen-triggered inflammation processes [40]. For example, miR-155 was induced in macrophages after activation of different inflammatory pathways [41] whereas decreased expression of miR-125a/b has been associated to inflammatory response against bacterial infection by activating macrophages, which release effector molecules (e.g. reactive oxygen, nitrogen intermediates) and inflammatory cytokines (e.g. IL1β, TNFα and IL6) [35, 42].

**Figure 6:**
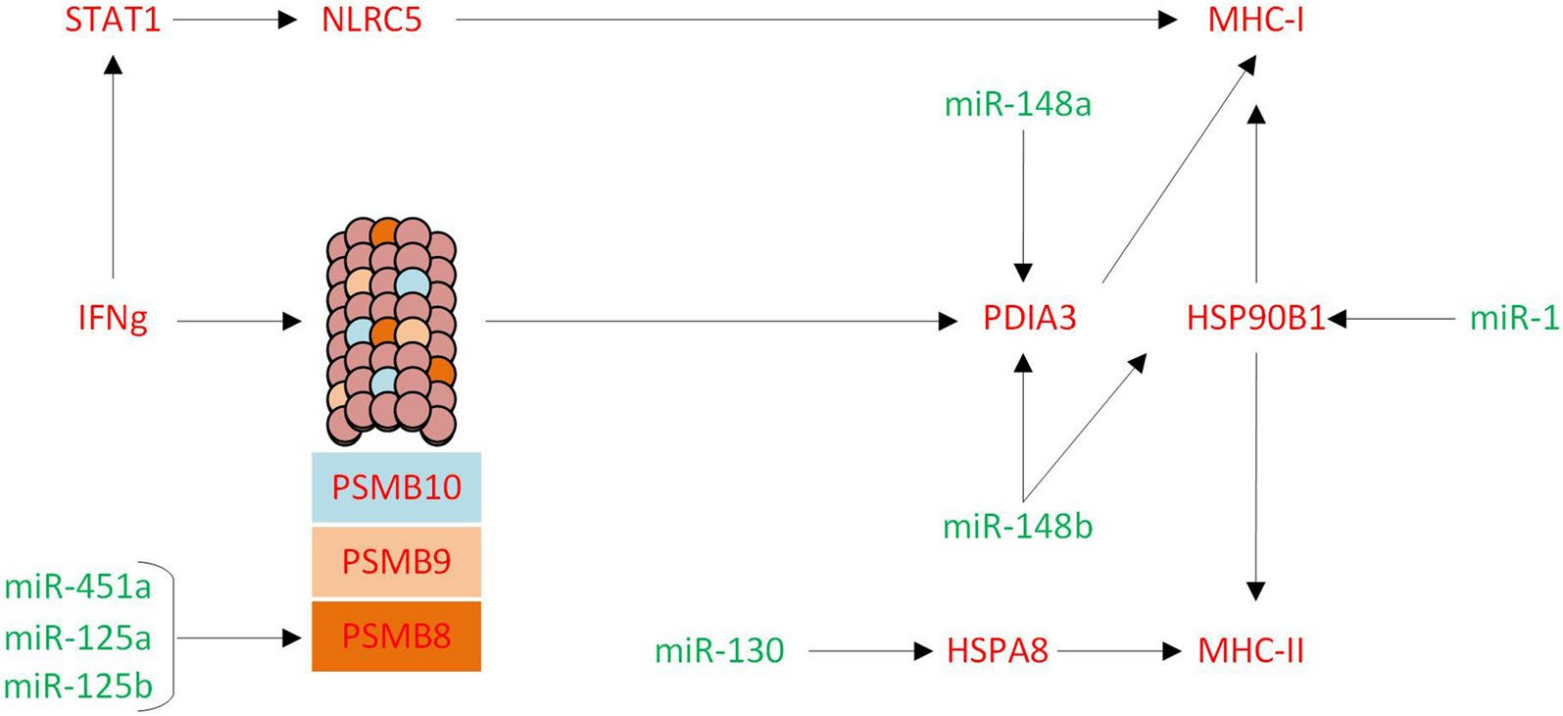
**Role of miRNA in the antigen cross presentation pathway.** Diagram showing how miR-451a, miR-125a/b, miR-130, miR-148a/b and miR-1 modulate various targets involved in antigenic presentation MHC-I and MHC-II in mesenteric lymph nodes during *Salmonella* Typhimurium infection.

The integrative analysis of miRNA and protein profiles allowed the identification of a large number of putative miRNA–mRNA interactions. Notably, based on our target prediction study and existing literature, most of the identified DE miRNA likely play important roles in regulation of immune-related pathways occurring in porcine MLN in response to *Salmonella* Typhimurium infection. For example, we predicted miRNA such as miR-125a/b as potential regulators of proteins involved in the MHC class I antigen presentation pathway such as PSMB8, PDIA3 and HSP90B1 [43] (Figure 6). The downregulation of miR-125 observed in this study could be regulating the expression of PSMB8, one of the three immunoproteasome components, together with PSMB9 and PSMB10 [44] (being the former also over-expressed in this study). In this work, we confirmed the direct interaction of miR-125 with PSMB8 supporting this theory. Similarly, miR-148a/b and miR-1 could be regulating MHC-class I components HSP90B1 and PDIA3, respectively. PDIA3 is part of the peptide loading complex, which is essential for the formation of the final antigen conformation and export from endoplasmic reticulum to cell surface [45]. HSP90B1 translocates antigens across the endosomal membrane into the cytosol [46]. Thus, our results suggest that a decreased expression of miR-125a/b, miR-148a/b and miR-1 could be contributing to regulate the *Salmonella* antigen presentation by MHC-I (Figure 6). Induction of the MHC-I gene expression was also evidenced in our experimental model of infection by over-expression of *NLRC5*, a specific transcriptional activator of the MHC-I expression, induced by IFNγ through STAT1 activation [47], whose coding genes were observed also over-expressed in this study. Cross-presentation of exogenous antigens from *Salmonella* origin via MHC-I molecules in the porcine MLN had already been suggested by us [8]. The broad pathways by which cross-presentation occurs have not been clearly elucidated yet, although in the most likely scenario, phagocytosed proteins reach cytosol by chaperone retrotranslocation mechanisms, and are subsequently degraded by proteasomes, binding MHC-I molecules and activating CD8+ T-cells [48–50]. Our target prediction analysis revealed miR-130a as a potential regulator of the gene that codes for HSPA8 protein (HSC70), which plays a central role in modulating antigen transport within cells to control MHC-II presentation [51]. This is in line with the knowledge that phagocytosis plays a traditional role in providing *Salmonella* antigens for MHC-II molecules to promote CD4(+) T cell recognition [52]. Together, our data indicate that miRNA might regulate MHC class I- and class II-dependent immune responses, which in turn activate a robust T-cell response for clearance of *Salmonella* infection. This agrees with the knowledge that some DE miRNA in this study such as miR-181a and miR-20a are known to target genes involved in regulation of T cell activation through T cell receptor induction [53].

Other mechanisms that control *Salmonella* spread into the host include cell death by apoptosis, autophagy and/or pyroptosis [8, 54]. In addition to our target prediction analysis, current literature supports that some of the DE miRNA in porcine MLN regulate genes involved in apoptosis (miR-21 and miR-125a) and autophagy (miR-30a, miR-204, miR-20a/b, miR-106 and miR-363) [55]. Our results indicate that apoptotic processes were inhibited in the infected tissue as a consequence of over-expression of miR-21 and downregulation of miR-125a. This expression pattern leads to inhibition of apoptosis by BAX repression, and subsequent BCL2 induction, as it has been previously described [56, 57]. The increased expression of *BCL2* observed in porcine MLN after infection is also in accordance with the ability of *Salmonella* to prevent apoptosis, as a strategy to provide survival advantage inside host cells [58]. From our mRNA study we can also conclude that no induction of autophagy is present in MLN after *Salmonella* Typhimurium infection. In fact, genes coding for LC3 (a potent autophagy inductor) and the autophagy negative regulator SQSTM1 [55] were observed down- and up-regulated, respectively. These results are in agreement with the observed decrease in expression of *ACTR3*, *YWHAZ*, *VIM* and *STMN1* genes, all of them involved in cytoskeletal rearrangements during the autophagic process [59]. Accordingly, we identified miR-363 and miR-92b as potential regulators of *ACTR3* and *YWHAZ* expression. Downregulation of miR-378a and miR-30b is likely implicated in downregulation of *VIM* expression. STMN1 is a protein involved in cytoskeletal rearrangements that functions by binding to tubulin αβ heterodimers (microtubules), sequestering them and preventing microtubule assembly. The role of STMN1 on immune cells is contradictive, because its downregulation has been described as critical for microtubule stabilization and macrophage activation [60] and affects T cells polarization [61]. In our study, we found that over-expression of miR-221 and miR-210 could lead to downregulation of STMN1. Recent works have shown that cytoskeletal rearrangements, particularly changes in actin conformation, are important for the entry of *Salmonella* into host cells [62, 63]. Although the over-expression of caspases has been related to the decrease of cytoskeletal proteins [64], previous studies have shown that in porcine MLN, *Salmonella* infection induces pyroptosis, a type of programmed cell death characterized by activation of caspase-1 and IL-1β production upon inflammatory antimicrobial responses [8]. In our study, both *CASP1* and *IL1β* genes were observed upregulated after *Salmonella* infection, while no significantly dysregulated miRNA were identified to play critical roles in regulation of these genes. Instead, target prediction analysis revealed interaction between miR-30d and PPID, a potent inductor of necroptosis, which is a programmed form of inflammatory cell death with an important function in host defense against intracellular bacteria [65].

In conclusion, our study provides novel evidence for functional molecular networks in MLN early after *Salmonella* Typhimurium infection. Integrative proteomic and miRNA analysis revealed that the miRNA–protein regulatory network is more complex than previously thought, highlighting that a single miRNA can regulate multiple target mRNA and vice versa. Although there are many different gene expression regulatory mechanisms in these biological processes, differential expression of miRNA could regulate the expression of proteins involved in networks critical for antigenic presentation, inflammatory response and cytoskeleton rearrangements through various signaling pathways during the acute phase of infection.

## Additional files

**Additional file 1.** **Primers used in this study.** Information of primers used in this study.

**Additional file 2.** **Summary of mapped reads to human and pig genomes.** Table showing input reads, reads after pre-processing (clean reads), and reads mapped to human and porcine mature miRNA’ databases.

**Additional file 3.** **miRNA profile of control and infected mesenteric lymph node.** Sequencing reads mapped to human (A) and porcine (B) miRNA databases, as well as miRNA exclusively mapped to each of those reference databases (C).

**Additional file 4.** **miRNA differentially expressed in porcine mesenteric lymph nodes after** ***Salmonella*** **Typhimurium infection.** miRNA differentially expressed in porcine mesenteric lymph nodes 2 days after *Salmonella* Typhimurium infection.

**Additional file 5.** **miRNA target protein prediction.** Prediction of targets from the differentially expressed miRNA (A) were integrated with proteomic data (B) and only negatively correlated miRNA-protein pairing (C) was used for further analysis.

## Abbreviations

MLN: mesenteric lymph nodes
3′UTR: 3′untranslated region
5′UTR: 5′untranslated region
cfu: colony forming units
dpi: days post-infection
MIQE: Minimum Information for Publication of Quantitative Real-Time PCR Experiments
IPA: Ingenuity Pathway Analysis
MREs: miRNA recognition elements
CHO: cells Chinese hamster ovary cells
FC: fold change
DE: differentially expressed
APC: antigen presenting cells
